# Serum cortisol and thyroid hormone concentrations and survival in foals born from mares with experimentally induced ascending placentitis

**DOI:** 10.1111/jvim.15758

**Published:** 2020-04-27

**Authors:** Vitória Müller, Ramiro E. Toribio, Katarzyna Dembek, Bruna S. S. Moraes, Mariana A. Mousquer, Bruna R. Curcio, Carlos E. W. Nogueira

**Affiliations:** ^1^ Departamento de Clínicas Veterinária, Faculdade de Medicina Veterinária Universidade Federal de Pelotas Pelotas Rio Grande do Sul Brazil; ^2^ Department of Veterinary Clinical Sciences College of Veterinary Medicine, The Ohio State University Columbus Ohio United States; ^3^ Department of Veterinary Clinical Sciences College of Veterinary Medicine, Iowa State University Iowa United States

**Keywords:** critical ill foals, rognostic marker, thyroxine, triiodothyronine

## Abstract

**Background:**

There are few publications on occurrence of nonthyroidal illness syndrome in foals and on the prognostic value of cortisol and thyroid hormone (TH) concentrations in newborn foals.

**Objectives:**

To determine serum cortisol and TH concentrations (total and free thyroxine: T_4_ and _F_T_4_; total and free triiodothyronine: T_3_ and _F_T_3_) in foals born from mares with placentitis, to determine their association with survival, and their use as prognostic markers.

**Animals:**

A cohort of 29 newborn foals comprising 5 Control, 14 Low‐risk, and 10 Sick foals were evaluated over the first week of life.

**Methods:**

In this prospective study foals born to mares with experimentally‐induced placentitis were assigned to Low‐risk or Sick groups while foals born to control mares were classified as Control based on clinical findings. Foals were also classified as Term (n = 13), Dysmature (n = 7), or Premature (n = 9), and survival rate was recorded. Serum cortisol and TH hormone concentrations were measured at 0, 12, 24, 48, and 168 hours of life.

**Results:**

Sick non‐surviving foals had lower (*P* < .05) T_3_ : cortisol ratio at 12 (3.68 ± 1.06 versus 18.58 ± 2.78), 24 (5.47 ± 2.34 versus 23.40 ± 3.82), and 48 (10.47 ± 6.29 versus 26.6 ± 2.90) hours of life when compared to Sick surviving foals and lower (*P* < .05) T_4_ : cortisol ratio at 12 (75.12 ± 21.71 versus 414.47 ± 58.47) and 24 hours (127.83 ± 55.21 versus 430.87 ± 80.31) after birth than Sick surviving foals.

**Conclusion and Clinical Importance:**

Placental infections can impair fetal thyroid function. Low T_3_ : cortisol and T_4_ : cortisol ratios seem to be good prognostic markers in newborn foals.

## 1 INTRODUCTION

Placentitis is one of the most important causes of fetoplacental unit impairment and can affect equine fetal maturation and postnatal development.^1^ Foals delivered by mares with placentitis can range from small sized foals with compromised organ function to full sized foals with no clinical and laboratory abnormalities, depending on both the time and the duration of the insult.^2^ Immaturity and sepsis are the main complications of placentitis to newborn foals and can lead to dysfunction of different organ systems.^3^, ^4^

In human neonates, disorders such as sepsis suppress the hypothalamic‐pituitary‐thyroid axis (HPTA), reducing thyroxine (T_4_), and triiodothyronine (T_3_) concentrations, leading to a condition known as nonthyroidal illness syndrome (NTIS).^5^, ^6^ The reduction in thyroid hormone (TH) concentrations possibly represents a physiological adaption to reduce metabolic activity to allow energy reallocation to respond to disease.^5^ Besides TH concentrations, T_3_ : T_4_ and _F_T_3_ : _F_T_4_ ratios have been used in human medicine to assess thyroid function and showed to be a useful aid in the diagnosis of NTIS as other thyroid dysfunctions.^7^

In neonates of different species, TH are important for thermoregulation in the first hours of life.^8^ In addition, these hormones act directly on cell metabolism and stimulation, which makes them essential for organs maturation and development.^8^, ^9^ Activation of the hypothalamic pituitary adrenal axis in the last weeks of pregnancy is essential for equine fetal tissue maturation and differentiation, but also for thyroid gland maturation and TH secretion.^10^, ^11^ The relation between adrenal and thyroid gland have been demonstrated in humans^12^ and rats.^13^ These studies suggested that excess of glucocorticoids can suppress the HPTA and that TH regulate adrenocortical function through changes in hepatic glucocorticoid metabolism.^12^, ^13^ Although the relation between adrenal and thyroid have not been deeply studied in foals, a relationship between cortisol and T_3_ concentrations have been demonstrated in the perinatal period.^10^ However, TH : cortisol ratios were not assessed before and could have clinical value.

There are few publications on NTIS in neonatal foals.^14^, ^15^, ^16^, ^17^ Furthermore, the information available is generally from hospital admissions, making it difficult to compare TH concentrations in sick and healthy age‐matched foals. Two studies demonstrated that sick foals have lower serum TH concentrations than healthy foals, suggesting that NTIS is prevalent in critically ill equine neonates. These studies also found that serum TH concentrations were lower in nonsurviving foals.^14^, ^17^

The aim of our study was to determine serum cortisol and TH concentrations (total thyroxine, T_4_; free thyroxine, _F_T_4_; total triiodothyronine, T_3_; and free triiodothyronine, _F_T_3_) in foals born from mares with experimentally‐induced ascending placentitis and to determine their association with survival. We hypothesized that (1) foals born to mares with experimentally induced ascending placentitis will have decreased serum cortisol and TH concentrations over the first week of life, (2) that these endocrine alterations will be associated with death, and (3) that cortisol and TH concentrations in the first hours of life will have prognostic value for survival.

## 2 MATERIALS AND METHODS

### 2.1 Animals

This observational cohort study was performed during the 2012‐2014 breeding seasons in Southern Brazil. Twenty‐nine foals were enrolled in our study from the time of birth to 7 days postpartum. These foals were also included in a parallel study, in which 46 foals were enrolled.^18^ However, only foals which serum samples were available were included in the present study. Five dams carried healthy gestations to term, while 24 dams were submitted to experimental induction of ascending placentitis through intracervical inoculation of 10^7^ colony‐forming units of *Streptococcus equi* subsp. *zooepidemicus* at 300 days of gestation, as previously described.^18^ Mares were assessed daily for the presence of vulvar discharge, early udder development and measurement of the combined thickness of uterus and placenta (CTUP), as described elsewhere.^19^ Of these mares, 100% (24/24) showed vulvar discharge, increased CTUP (>12 mm) and 20.8% (5/24) presented early udder development at 48 hours postbacterial inoculation. Mares were started on medical treatment at 48 hours postbacterial inoculation with flunixin meglumine (1.1 mg/kg, IV, q24h for 10 days; Flumedin, Ouro Fino Saude Animal, São Paulo, Brazil) and sulfamethoxazole and trimethoprim (30 mg/kg, IV, q12h for 10 days; Trissulfim, Ouro Fino Saude Animal, São Paulo, Brazil). Furthermore, 8 mares (8/24) were supplemented with long‐acting altrenogest (0.088 mg/kg, IM, 2 administrations q7 days; Altrengest, Botupharma, São Paulo, Brazil), 5 mares (5/24) with estradiol cypionate (10 mg/mare, IM, 3 administrations q3 days; E.C.P., Zoetis, São Paulo, Brazil), and 5 mares (5/24) received both drugs as described elsewhere.^18^

### 2.2 Clinical assessment of the foals

All foalings were closely monitored. Foals had gestation duration recorded in days and were submitted to physical exams, evaluation of righting reflexes, total blood count and measurement of creatinine, urea, glucose and blood lactate immediately after birth. Foals born to mares with normal gestations showed normal adaptive behavior (sternal recumbency < 5 minutes, suckling reflex < 20 minutes, and stand < 1 hour), no evidence of immaturity, appropriate serum immunoglobulin G concentrations (IgG > 8.0 g/L), normal physical exams and total blood counts^20^ during the first week of life and were assigned to the Control group (n = 5). Foals delivered by mares submitted to experimental induction of ascending placentitis were classified as Low‐risk or Sick at 12 hours after birth, when the sepsis scores were calculated.^21^ Foals with normal adaptive behavior, physical exams, serum IgG concentrations > 800 mg/dL, unremarkable CBCs, and sepsis score ≤ 10 were assigned to Low‐risk group (n = 14), while foals that needed assistance to nurse and showed abnormal adaptive behavior, physical exams, CBCs, sepsis scores ≥ 11, or all were assigned to the Sick group (n = 10). In Sick group, 7 foals were septic while 3 foals had sepsis score ≤ 10 but were considered sick because of, abnormal adaptive behavior and alterations on clinical parameters, CBCs, and biochemical results.

Foals delivered with ≥320 days of gestation and showing no physical signs of immaturity were classified as Term (n = 13). Foals born with ≥320 days of gestation and showing physical signs consistent with immaturity (small body size, short and shiny hair coat, prominent rounded head, periarticular laxity, droopy ears and weakness) were classified as Dysmature (n = 7), while those delivered with <320 days of gestation and showing physical signs consistent with immaturity were classified as Premature (n = 9), according to the classification described elsewhere.^3^ In the Low‐risk group, 1 foal was considered premature and 6 were considered dysmature. All foals in the Sick group had physical findings consistent with immaturity. Eight were considered premature and 2 dysmature.

Foals from the Sick group received ampicillin sodium (20 mg/kg, IV, q8h; Ampicilina Veterinária, Vetnil, São Paulo, Brazil) and flunixin meglumine (0.5 mg/kg, IV, q12h; Flumedin, Ouro Fino Saude Animal, São Paulo, Brazil) and fluid therapy as needed. Treatments started at 24 hours after birth. Survival rate was recorded until 60 days of age. Six foals from the sick group did not survive. Two of them had to be euthanized due the grave prognosis.

### 2.3 Hormonal analysis

Foals had blood collected from the external jugular vein immediately after birth (0 hours), and at 12, 24, 48 hours and 7 days (168 hours) postpartum. Samples were allowed to clot at room temperature for 20 minutes, centrifuged at 600*g* for 10 minutes and immediately stored at −20°C until further analysis. Serum concentrations of total thyroxine (T_4_), free thyroxine (_F_T_4_), total triiodothyronine (T_3_), and free triiodothyronine (_F_T_3_) were determined by enzyme‐linked immunosorbent assay (MP Biomedicals LLC, New York), whereas cortisol was determined by radioimmunoassay (MP Biomedicals LLC). The sensitivity and specificity of the kits used are described in Table 1.

**TABLE 1:** Technique, catalog number, standard range, sensitivity, and specificity of the assays used to measure hormone concentrations in foals

Hormone	Technique	Catalog number	Standard range*	Sensitivity*	Specificity	
Cortisol	RIA	07‐221102	0.01‐100 μg/dL	0.0017 μg/mL	Cortisol (100%), prednisolone (45.6%), 11‐desoxycortissol (12.3%), corticosterone (5.5%), prednisone (2.7%), cortisone (2.1%), 17α‐hydroxyprogesterone (1%), progesterone (0.25%), and testosterone (<0.01%).	
T3	ELISA	05BC‐1005	0.00‐15.36 nmol/L	0.31 nmol/L	Triiodo‐l‐thyronine (100%), triiodo‐d‐thyronine (90%), l‐thyroxine (0%), d‐thyroxine (16%), triiodothyroacetic acid (<8%), monoiodotyrosine (0%), diiodotyrosine (0.08%), methimazole (0.05%), 5,5′‐diphenylhydantion (0%), phenylbutazone (0.001%), salicylic acid (0%), and acetylsalicylic acid (0%).	
FT3	ELISA	07BC‐1006	0.0‐321.80 pmol/L	0.08 pmol/L	I‐triiodothyronine (100%), I‐thyronine (<0.0002%), iodothyrosine (<0.0001%), diiodothyrosine (<0.0001%), phenylbutazone (<0.0001%), and sodium salicylate (<0.0001%).	
T4	ELISA	07BC‐1004	0.0–321.80 nmol/L	6.44 nmol/L	l‐Thyroxine (92.1%), d‐thyroxine (100%), triiodo‐l‐thyronine (22.6%), triiodo‐d‐thyronine (32%), triiodothyroacetic acid (0%), monoiodotyrosine (0%), diiodotyrosine (0%), methilmazole (0%), 5,5′‐diphenylhydantion (0%), phenylbutazone (0%), 6‐n‐propyl‐2‐triouracil (0%), salicylic acid (0%), and acetylsalicylic acid (0%).	
FT4	ELISA	07BC‐1008	0.0‐90.30 pmol/L	0.65 pmol/L	Not available	

Abbreviation: RIA, radioimmunoassay.

Manufacturer: MP Biomedicals (Orangeburg, New York).

### 2.4 Statistical analysis

All variables were tested for normality with the Shapiro‐Wilk test. Hormone concentrations and T_3_ : T_4_, _F_T_3_ : _F_T_4_, T_3_ : cortisol and T_4_ : cortisol ratios were compared among age‐matched foals (at birth and 12, 24, 48 and 168 hours after birth) and between groups by 2‐way repeated measures ANOVA and post hoc comparisons were made by Tukey test. The area under the curve (AUC) was calculated by the trapezoidal method for TH concentrations for Sick surviving and Sick nonsurviving foals and comparisons were carried out by *t*‐test. Hormone concentrations are described as mean ± SEM. Statistical analysis were performed by commercial software (SigmaStat 3.5, Systat Software, San Jose, California). Significance was set at *P* < .05.

## 3 RESULTS

**FIGURE 1:**
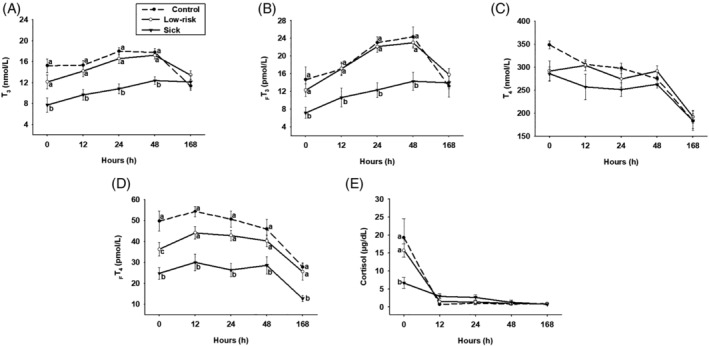
Thyroid hormone, A‐D, and cortisol, E, serum concentrations in foals assigned to Control (n = 5), Low‐risk (n = 14), and Sick (n = 10) group. Statistical difference (*P* < .05) among groups is represented by different letters at each time point. T_3_, total triiodothyronine; _F_T_3_, free triiodothyronine; T_4_, total thyroxine; _F_T_4_, free thyroxine

Serum T_3_ and _F_T_3_ concentrations were lower in Sick compared to Control and Low‐risk foals at 0, 12, 24, and 48 hours after birth (Figure 1; *P* < .05), but not different at 7 days of age. Serum T_4_ concentrations were not different among groups at any time point (*P* > .05). However, serum _F_T_4_ concentrations were lower in Sick than in Control and Low‐risk foals from 0 to 168 hours after birth (Figure 1; *P* < .05). Sick foals had decreased concentrations of cortisol than Control and Low‐risk foals at 0 hours (Figure 1; *P* < .01). Hormone ratios were not different among Control, Low‐risk, and Sick groups (*P* > .05).

**FIGURE 2:**
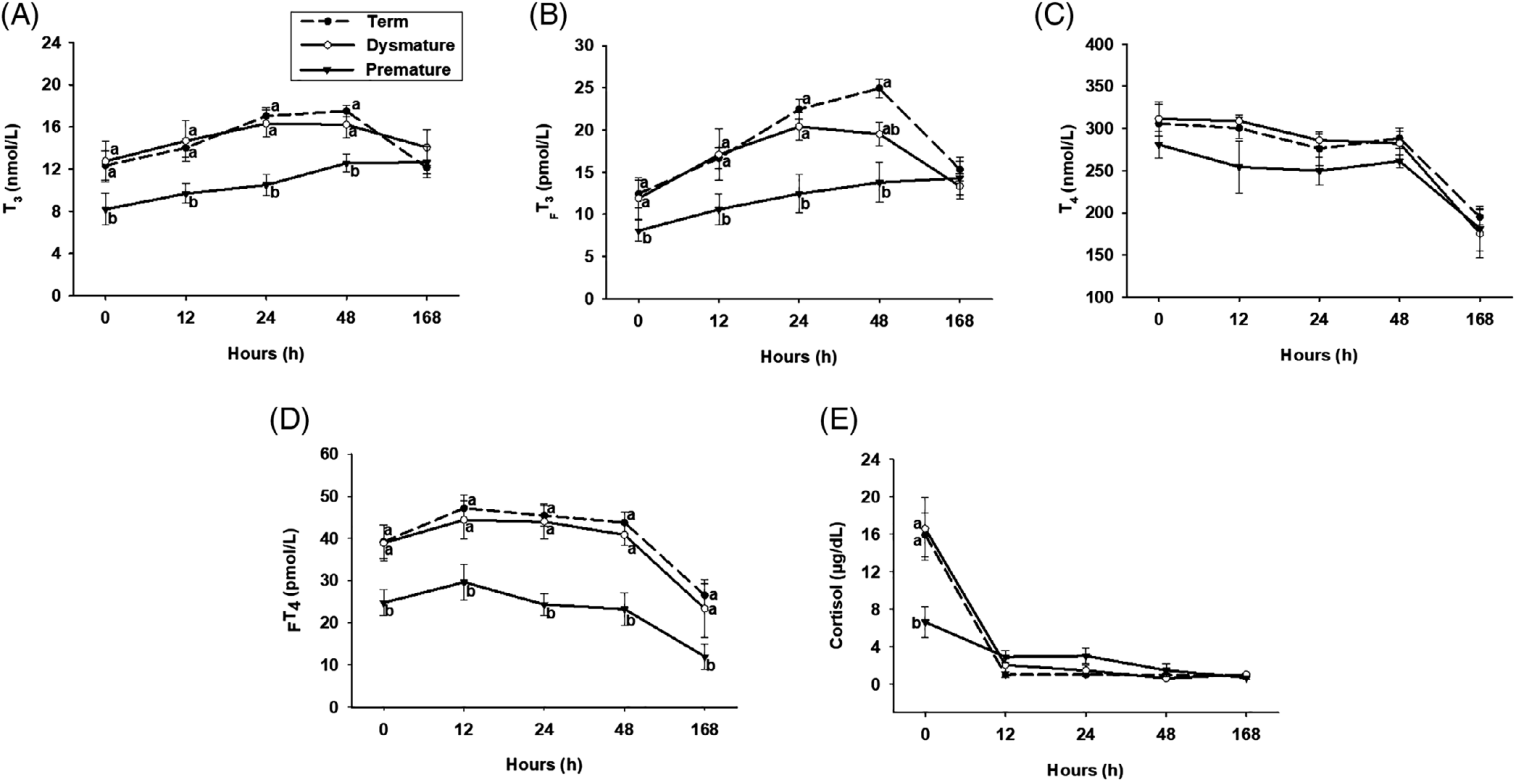
Thyroid hormone, A‐D, and cortisol, E, serum concentrations in Term (n = 13), Dysmature (n = 7), and Premature (n = 9) foals. Statistical difference (*P* < .05) among groups is represented by different letters at each time point. T_3_, total triiodothyronine; _F_T_3_, free triiodothyronine; T_4_, total thyroxine; _F_T_4_, free thyroxine

**FIGURE 3:**
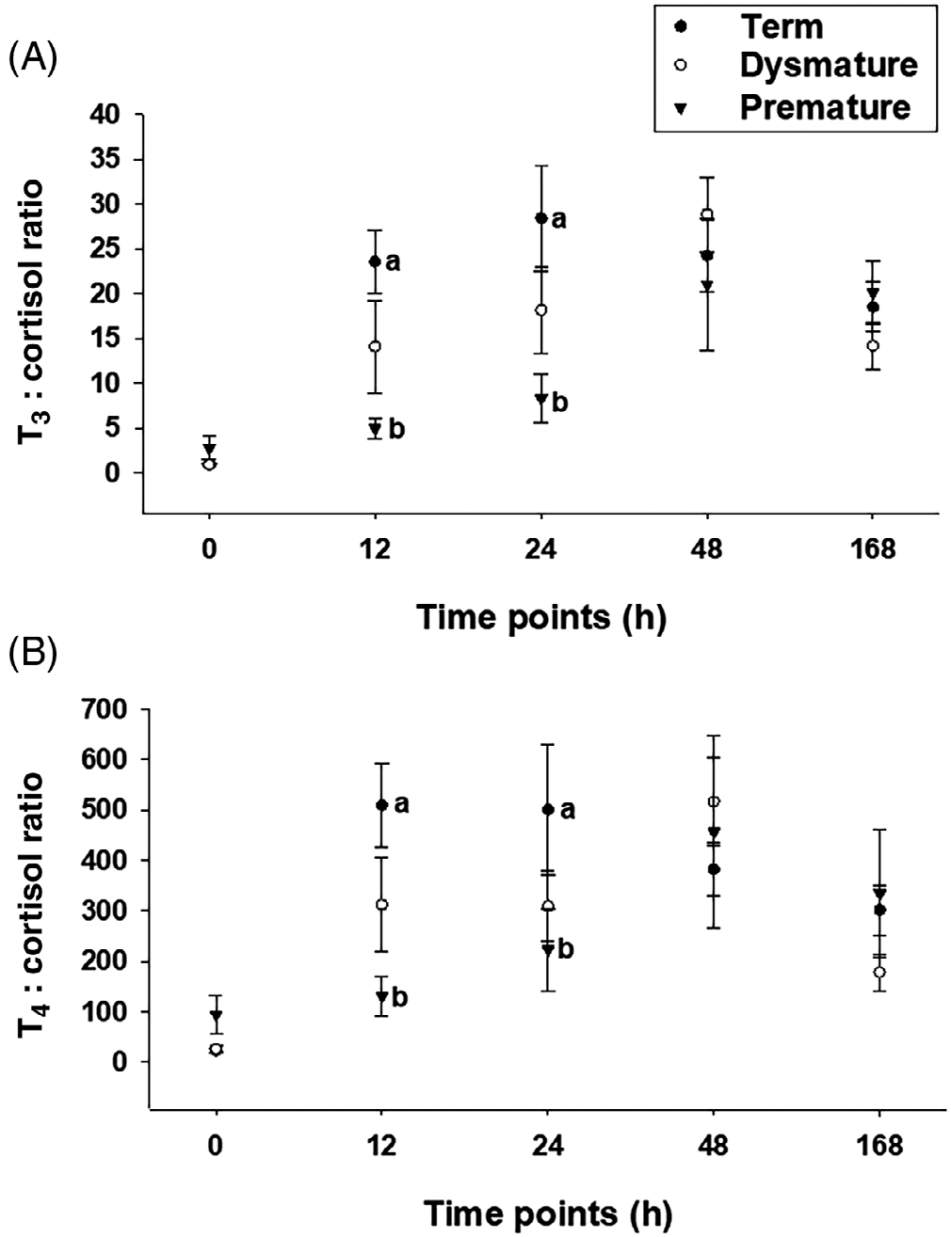
T_3_ : cortisol, A, and T_4_ : cortisol, B, ratios in Term (n = 13), Dysmature (n = 7), and Premature (n = 9) foals. Statistical difference (*P* < .05) among groups is represented by different letters at each time point. T_3_, total triiodothyronine; T_4_, total thyroxine

Premature foals had lower T_3_ and _F_T_3_ serum concentrations than Term foals at 0, 12, 24, 48 hours of life (*P* < .01), and lower than dysmature at 12 and 24 hours after birth (*P* < .05). Serum T_4_ concentrations were not different among groups. However, serum _F_T_4_ concentrations were lower in Premature compared to Term and Dysmature foals at all time points (*P* < .05). Premature foals had lower cortisol concentrations than Term and Dysmature foals at 0 hours (Figure 2; *P* < .01). Premature foals had lower T_3_ : cortisol and T_4_ : cortisol ratios than Dysmature and Term foals at 12 and 24 hours after birth (Figure 3; *P* < .035).

**FIGURE 4:**
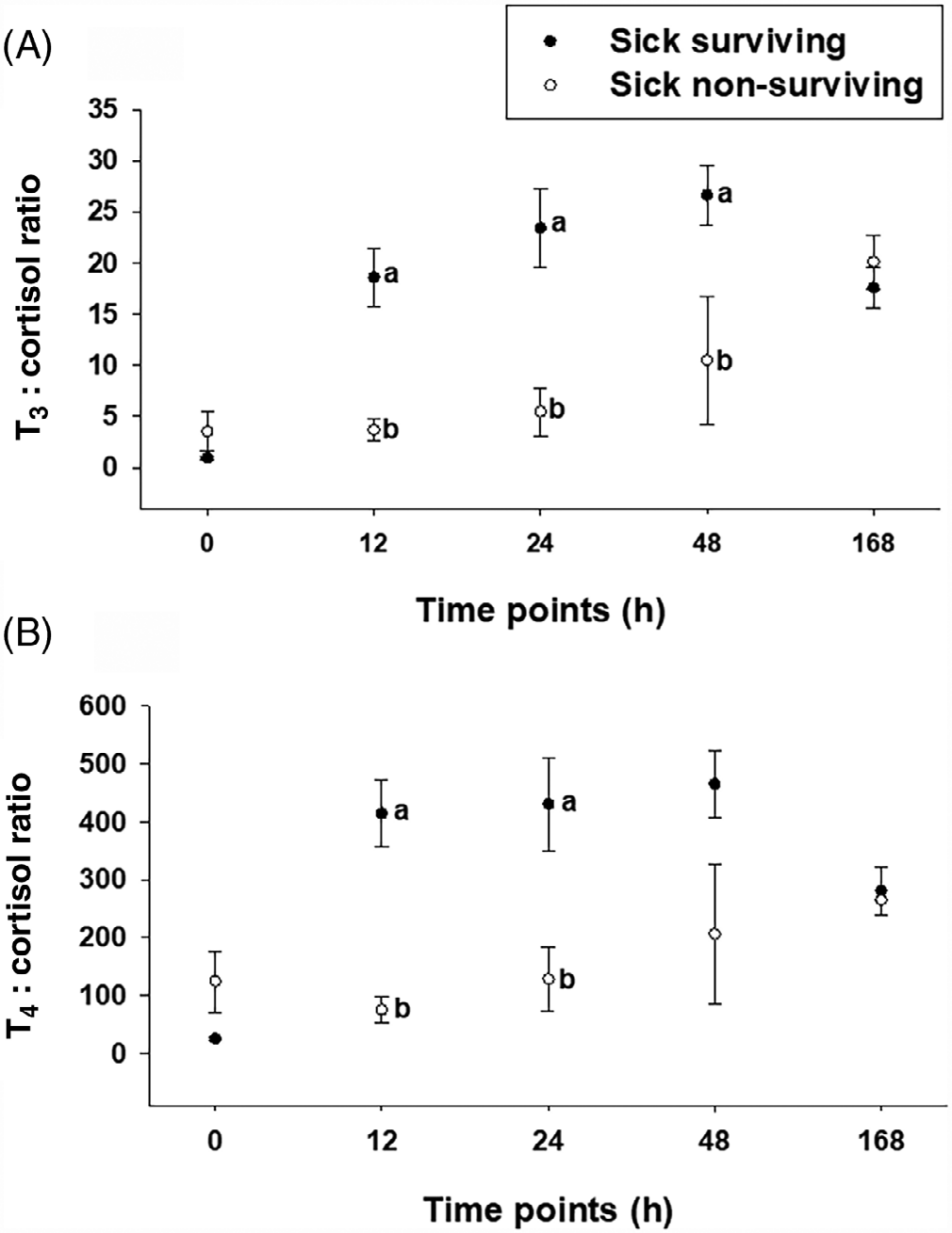
T_3_ : cortisol, A, and T_4_ : cortisol, B, ratios in surviving (n = 4) and nonsurviving (n = 6) Sick foals. Statistical difference (*P* < .05) among groups is represented by different letters at each time point. T_3_, total triiodothyronine; T_4_, total thyroxine

Serum T_3_, _F_T_3_, T_4_, _F_T_4_, and cortisol concentrations were not different between surviving and nonsurviving foals (Table 2; *P* > .05). Sick nonsurviving foals had lower T_3_ : cortisol ratio from 12 to 48 hours of life and lower T_4_ : cortisol ratio at 12 and 24 hours after birth than Sick surviving foals (Figure 4; *P* = .031). The AUC for TH was not different between Sick surviving and Sick nonsurviving foals (*P* > .05).

**TABLE 2:** Serum cortisol and thyroid hormone concentrations in surviving (n = 4) and nonsurviving (n = 6) Sick foals

Hormone	Group	Time point (hours)				
		0	12	24	48	168
Cortisol (μg/dL)	Surviving (n = 4)	8.49 ± 2.32	9.59 ± 1.73	11.26 ± 1.23	12.11 ± 0.99	10.20 ± 0.90
	Nonsurviving (n = 6)	7.23 ± 1.87	9.72 ± 1.35	10.52 ± 1.38	12.78 ± 1.24	15.05 ± 0.41
T_3_ (nmol/L)	Surviving (n = 4)	7.15 ± 2.68	10.67 ± 3.69	15.83 ± 2.60	16.48 ± 2.70	13.37 ± 2.42
	Nonsurviving (n = 6)	7.21 ± 1.31	10.60 ± 2.86	10.60 ± 1.84	12.12 ± 2.93	14.98 ± 0.19
_F_T_3_ (pmol/L)	Surviving (n = 4)	311.86 ± 17.62	298.26 ± 17.60	270 ± 10.57	257.59 ± 12.05	172.51 ± 34.37
	Nonsurviving (n = 6)	267.93 ± 19.43	299.36 ± 42.29	238.58 ± 23.24	269.33 ± 11.09	199.83 ± 19.45
T_4_ (nmol/L)	Surviving (n = 4)	27.24 ± 3.72	32.36 ± 6.16	32.86 ± 5.71	30.75 ± 5.95	12.09 ± 2.33
	Nonsurviving (n = 6)	23.12 ± 4.12	28.16 ± 5.58	22.04 ± 2.65	25.84 ± 6.61	13.88 ± 1.59
_F_T_4_ (pmol/L)	Surviving (n = 4)	9.28 ± 2.32	2.08 ± 1.18	1.49 ± 0.97	0.41 ± 0.10	0.72 ± 0.21
	Nonsurviving (n = 6)	4.95 ± 1.76	3.61 ± 0.84	3.45 ± 0.93	2.53 ± 1.20	0.76 ± 0.08

*Note:* Results expressed as mean ± SE. Statistical differences were not detected among groups (*P* > .05).

Abbreviations: T_3_, total triiodothyronine; _F_T_3_, free triiodothyronine; T_4_, total thyroxine; _F_T_4_, free thyroxine.

## 4 DISCUSSION

In our study, sick foals born from mares with experimentally induced placentitis had lower concentrations of T_3_, _F_T_3_, and _F_T_4_ than healthy control and Low‐risk foals at birth and during the first week of life, suggesting that these foals developed NTIS in response to intrauterine disease. Similar results were found in that sick foals up to 6 hours of life had lower concentrations of T_4_ and T_3_ than healthy foals.^15^

The mechanism by which sick foals delivered by mares with placental impairment had abnormally low TH concentrations was not elucidated in the present study. However, we demonstrated that premature foals had lower cortisol concentrations 0 hours postpartum and lower TH concentrations in the majority of the time points evaluated. The fact that most (8/10) foals in the Sick group were premature indicates that fetal maturation could have impaired thyroid gland function or that low TH concentrations interfered with fetal maturation. A correlation between T_3_ and cortisol concentrations has been documented in equine fetuses, suggesting that increased adrenocortical activity and subsequent cortisol release few days before birth promotes thyroid gland maturation and TH secretion.^10^ In the same study, premature foals had lower concentrations of cortisol and T_3_ than term foals, whereas dysmature foals had lower concentrations of T_3_ than term foals, but higher than premature foals.^10^ These findings were similar to the ones from the study reported here.

Sick foals from the present study had lower TH serum concentrations at birth and likely in late gestation, supporting impaired TH synthesis from placental and fetal disease, which is consistent with NTIS. Different mechanisms have been proposed in the pathogenesis of NTIS, including abnormal concentrations of inflammatory cytokines and leptin.^5^ A number of inflammatory cytokines (IL‐1β, IL‐6, IL‐8, IL‐15, IL‐18, and IFN‐γ) have been shown to be elevated in mares with naturally occurring and experimentally induced placentitis.^22^, ^23^ In addition, several cytokines are involved in the pathophysiology of sepsis in neonatal foals.^24^ There is evidence that the tumor necrosis factor alpha (TNF‐α), IL‐1, IL‐6, and IL‐10 have negative effects on thyroid‐stimulating hormone (TSH) release, on the expression of deiodinases that convert T_4_ to T_3,_ and decrease TH binding to serum proteins.^25^ In addition, inflammatory cytokines inhibit genes involved in the metabolism of TH.^26^, ^27^ Although inflammatory cytokines were not assessed in mares and foals from our study, they might have contributed to the reduction of TH in sick foals. The fact that some foals have experienced an inflammatory process in uterus, but were considered Low‐risk foals and had normal TH levels lead us to believe that some mares had a mild inflammatory response to infection or responded more efficiently to treatment.

In the present study, we found no association between serum hormone concentrations and foal survival. Similar results were found in another study with foals affected with perinatal asphyxia syndrome in which TH concentrations were not associated with survival.^16^ Other foal studies have found association between low TH concentrations and death.^14^, ^17^ In a study in which a single blood sample was collected to assess TH concentration in foals that were between 1 and 192 hours old, septic nonsurviving foals had lower levels of T_3_, _F_T_3_, and _F_T_4_ than septic surviving foals.^14^ Other study that evaluated TH levels in foals that were 24 to 36 hours old demonstrated that sick nonsurviving foals had decreased levels of _F_T_3_ and premature nonsurviving foals had decreased levels of _F_T_3_ and _F_T_4._ ^17^ However, in the foals of our study, TH concentrations were measured multiple times over a 1‐week period since the physiological decrease in TH concentrations in newborn foals suggests that for proper TH result interpretation, they should be compared to age‐matched healthy foals.

The relationship between adrenal and thyroid gland have been proposed in different species.^10^, ^12^, ^13^ However, cortisol : TH ratios have not been assessed before. Interestingly, although in our study TH concentrations were not related to survival, T_3_ : cortisol and T_4_ : cortisol ratios were significantly lower in sick nonsurviving foals than in sick surviving foals. This information suggests that T_3_ : cortisol and T_4_ : cortisol ratios could have prognostic value in critically ill neonatal foals.

## 5 CONCLUSION

Placental infection can impair fetal thyroid function. Serum cortisol and TH concentrations changed according to the clinical condition and maturity of the foals. Sick foals delivered by mares with experimentally‐induced ascending placentitis had decreased concentrations of T_3_, _F_T_3_, and _F_T_4_ over the first week of life suggesting that these foals had NTIS. TH serum concentrations were not associated with neonatal death in our study. However, T_3_ : cortisol and T_4_ : cortisol ratios seem to be good prognostic markers of disease severity in the first hours of life.

## CONFLICT OF INTEREST DECLARATION

Authors declare no conflict of interest.

## OFF‐LABEL ANTIMICROBIAL USE DECLARATION

Authors declare no off‐label use of antimicrobials.

## INSTITUTIONAL ANIMAL CARE AND USE COMMITTEE (IACUC) DECLARATION

All procedures carried out in the present study were approved by the Ethical Committee on Animal Experimentation of the Federal University of Pelotas under protocol #4750.

## HUMAN ETHICS APPROVAL DECLARATION

Authors declare human ethics approval was not needed for our study.

## References

[1] Morresey PR . Prenatal and perinatal indicators of neonatal viability. Clin Tech Equine Pract. 2005;4:238‐249. 10.1053/j.ctep.2005.07.005.

[2] Bain FT . Management of the foal from the mare with placentitis: a clinician's approach. 50th Annu. Conv. Am. Assoc. Equine Pract.; 2004; Denver.

[3] Rossdale PD , Ousey JC , Silver M , et al. Studies on equine prematurity VI: guidelines for assessment of foal maturity. Equine Vet J. 1984;16:300‐302. 6090120 10.1111/j.2042-3306.1984.tb01931.x

[4] Sanchez LC . Equine neonatal sepsis. Vet Clin North Am Equine Pract. 2015;21:273‐293. 10.1016/j.cveq.2005.04.007. 16051050

[5] Farwell AP . Nonthyroidal illness syndrome. Curr Opin Endocrinol Diabetes Obes. 2013;20:478‐484. 10.1097/01.med.0000433069.09294.e8. 23974778

[6] Silva MHBN , Araujo MCK , Diniz EMA , et al. Nonthyroidal illnesses syndrome in full‐term newborns with sepsis. Arch Endocrinol Metab. 2015;59:528‐534. 10.1590/2359-3997000000111. 26677087

[7] Laurberg P . Mechanisms governing the relative proportions of thyroxine and 3,5,3′‐triiodothyronine in thyroid secretion. Metabolism. 1984;33:379‐392. 6369072 10.1016/0026-0495(84)90203-8

[8] Irvine CHG . Hypothyroidism in the foal. Equine Vet J. 1984;16:302‐306. 10.1111/j.2042-3306.1984.tb01932.x. 6383812

[9] Toribio RE . Endocrine dysregulation in critically ill foals and horses. Vet Clin North Am—Equine Pract. 2011;27:35‐47. 10.1016/j.cveq.2010.12.011. 21392652

[10] Silver M , Fowden AL , Knox J , Ousey J , Cash R , Rossdale PD . Relationship between circulating tri‐iodothyronine and cortisol in the perinatal period in the foal. J Reprod Fertil Suppl. 1991;44:619‐626. 1665521

[11] Fowden AL , Forhead AJ , Ousey JC . Endocrine adaptations in the foal over the perinatal period. Equine Vet J. 2012;44:130‐139. 10.1111/j.2042-3306.2011.00505.x. 22594041

[12] Samuels MH , McDaniel PA . Thyrotropin levels during hydrocortisone infusions that mimic fasting induced cortisol elevations: a clinical research center study. J Clin Endocrinol Metab. 1997;82:3700‐3704. 9360528 10.1210/jcem.82.11.4376

[13] Johnson EO , Calogero AE , Konstandi M , Kamilaris TC , la Vignera S , Chrousos GP . Effects of short‐ and long‐duration hypothyroidism on hypothalamic‐pituitary‐adrenal axis function in rats: in vitro and in situ studies. Endocrine. 2012;42:684‐693. 22695985 10.1007/s12020-012-9714-z

[14] Himler M , Hurcombe SDA , Griffin A , et al. Presumptive nonthyroidal illness syndrome in critically ill foals. Equine Vet J. 2012;44:43‐47. 10.1111/j.2042-3306.2011.00480.x. 22594025

[15] Panzani S , Comin A , Galeati G , et al. How type of parturition and health status influence hormonal and metabolic profiles in newborn foals. Theriogenology. 2012;77:1167‐1177. 10.1016/j.theriogenology.2011.10.023. 22153270

[16] Pirrone A , Panzani S , Govoni N , Castagnetti C , Veronesi MC . Theriogenology thyroid hormone concentrations in foals affected by perinatal asphyxia syndrome. Theriogenology. 2013;80:624‐629. 10.1016/j.theriogenology.2013.06.003. 23849257

[17] Breuhaus BA . Thyroid function and dysfunction in term and premature equine neonates. J Vet Intern Med. 2014;2014:1301‐1309. 10.1111/jvim.12382. PMC4857961 24934827

[18] Curcio BR , Canisso IF , Pazinato FM , et al. Estradiol cypionate aided treatment for experimentally induced ascending placentitis in mares. Theriogenology. 2017;102:98‐107. 10.1016/j.theriogenology.2017.03.010. 28755579

[19] Bucca S , Fogarty U , Collins A , Small V . Assessment of feto‐placental well‐being in the mare from mid‐gestation to term: transrectal and transabdominal ultrasonographic features. Theriogenology. 2005;64:542‐557. 15993936 10.1016/j.theriogenology.2005.05.011

[20] Koterba AM , Drummond WH , Kosch PC . Equine Clinical Neonatology. 1st ed. Philadelphia, Pennsylvania: Lea & Febiger; 1990.

[21] Brewer BD , Koterba AM . Development of a scoring system for the early diagnosis of equine neonatal sepsis. Equine Vet J. 1988;20:18‐22. 10.1111/j.2042-3306.1988.tb01445.x. 3366100

[22] Lyle SK . The Relationship Between Pro‐inflammatory Cytokines, Prostaglandins, and Fetal Hypothalamic‐Pituitary‐Adrenal Axis Activation in Mares with Infective Pre‐term Delivery. Louisiana: Louisiana State University; 2008.

[23] Leblanc MM , Giguère S , Lester GD , et al. Relationship between infection, inflammation and premature parturition in mares with experimentally induced placentitis. Equine Vet J. 2012;44:8‐14. 10.1111/j.2042-3306.2011.00502.x. 22594019

[24] McKenzie HC III , Furr MO . Equine neonatal sepsis: the pathophysiology of severe inflammation and infection. Compend Contin Educ Pract Vet. 2014;23:661‐672.

[25] Boelen A , Kwakkel J , Fliers E . Beyond low plasma T3: local thyroid hormone metabolism during inflammation and infection. Endocr Rev. 2011;32:670‐693. 10.1210/er.2011-0007. 21791567

[26] Tominaga T , Yamashita S , Nagayama Y , et al. Interleukin 6 inhibits human thyroid peroxidase gene expression. Acta Endocrinol. 1991;124:290‐294. 2011918 10.1530/acta.0.1240290

[27] Tang KT , Braverman LE , DeVito WJ . Tumor necrosis factor‐α and interferon‐^γ^ modulate gene expression of type I 5′‐deiodinase, thyroid peroxidase, and thyroglobulin in FRTL‐5 rat thyroid cells. Endocrinology. 1995;136:881‐888. 7867596 10.1210/endo.136.3.7867596

